# A path to practical Solar Pumped Lasers via Radiative Energy Transfer

**DOI:** 10.1038/srep14758

**Published:** 2015-10-05

**Authors:** Philip D. Reusswig, Sergey Nechayev, Jennifer M. Scherer, Gyu Weon Hwang, Moungi G. Bawendi, Marc. A. Baldo, Carmel Rotschild

**Affiliations:** 1 Department of Electrical Engineering and Computer Science, Massachusetts Institute of Technology, 77 Massachusetts Avenue, Cambridge, MA 02139, USA; 2 Department of Mechanical Engineering and Russell Berrie Nanotechnology Institute, Technion-Israel Institute of Technology, Haifa 32000, Israel; 3 Department of Chemistry, Massachusetts Institute of Technology, 77 Massachusetts Avenue, Cambridge, MA 02139, USA

## Abstract

The optical conversion of incoherent solar radiation into a bright, coherent laser beam enables the application of nonlinear optics to solar energy conversion and storage. Here, we present an architecture for solar pumped lasers that uses a luminescent solar concentrator to decouple the conventional trade-off between solar absorption efficiency and the mode volume of the optical gain material. We report a 750-μm-thick Nd^3+^-doped YAG planar waveguide sensitized by a luminescent CdSe/CdZnS (core/shell) colloidal nanocrystal, yielding a peak cascade energy transfer of 14%, a broad spectral response in the visible portion of the solar spectrum, and an equivalent quasi-CW solar lasing threshold of 23 W-cm^−2^, or approximately 230 suns. The efficient coupling of incoherent, spectrally broad sunlight in small gain volumes should allow the generation of coherent laser light from intensities of less than 100 suns.

The conversion of broadband solar radiation into coherent and narrow-band laser radiation has attracted attention in recent years due to its potential applicability in solar energy conversion. Solar pumped lasers (SPL) can be used to drive reactions that store solar energy in chemical form^1^, or perform efficient wavelength conversion of sub-bandgap sunlight, potentially increasing the efficiency of solar cells beyond the single junction limit^2^. The first solar pumped laser was reported by RCA Laboratories in 1963 based on a Dy^2+^:CaF_2_ crystalline system^3^. Since then many advances in optical gain media and solar collector design have occurred^1^,^4^,^5^,^6^,^7^,^8^. The most common optical gain media used in solar pumped lasers today is the rare earth ion neodymium (Nd^3+^) due to its natural four-level system, high emission cross-section, and long excited state lifetime. Despite these favorable optical gain properties, neodymium is not an ideal solar absorber due to poor spectral overlap with sunlight and low peak absorption coefficients, of <10 cm^−1^ at λ ~ 800 nm that necessitate large optical gain media volumes. While larger gain volumes result in increased solar pump absorption, the equivalent increase in mode volume cancels out the increase in excitation density of the mode that is essential for reaching population inversion. Consequently, state-of-the-art solar pumped lasers operate above 1000 suns. Nd^3+^:YAG laser rods, for example, operate at power thresholds of 275 W-cm^−2^ utilizing large Fresnel lenses to concentrate sunlight^4^. Nd^3+^:YAG ceramics sensitized by Cr^3+^ ions have broad absorption bands in the visible spectrum, and solar lasing thresholds of 160 W-cm−2 21,5. S. Mizuno *et al.* demonstrated passive cooling of a solar pumped laser through the use of a small volume Nd^3+^-doped glass fiber operating at a solar lasing threshold of 370 W-cm−2 8. Finally, Nd-Doped Active fiber sensitized by Rhodamine solution was demonstrated to induce gain when pumped by Xenon lamp. Yet, the gain per input power was far from lasing due to the high self-absorption and poor spectral overlap^9^. In this work we use cascade energy transfer^10^ to decouple solar pump absorption and optical gain, thereby enabling solar pumped lasers with efficient solar absorption in small mode volumes. A schematic of the optical energy transfer process is shown in Fig. 1. The scheme begins with incident sunlight absorbed by a layer of luminescent colloidal semiconductor nanocrystals. These are ideal solar down-shifting materials due to their stability^11^, narrow photoluminescence spectrum, and high photoluminescence quantum yield, and broadband absorption coefficients of >10^5^ cm^−1^ above an exciton absorption line that is tunable through size and material composition^12^. The incident light absorbed by the nanocrystals is then re-emitted as photoluminescence. As in the operation of a luminescent solar concentrator (LSC)^13^,^14^, a fraction of the emitted photoluminescence is trapped within the waveguide formed by the optical gain media and its nanocrystal coating. The fraction of emitted photons that is subject to total internal reflection is known as the trapping efficiency. A function of the refractive index^14^, the trapping efficiency of wave-guided photoluminescence is approximately 75% for typical dopant hosts such as polymers and glasses with n = 1.5.

The semiconductor nanocrystals are chosen such that their photoluminescence wavelength matches an absorption peak of the optical gain medium (Fig. 2b). As the photoluminescence propagates in the waveguide, energy is transferred to the optical gain media through absorption. The optical concentration achieved in the waveguide can be increased by reducing its thickness, thereby enabling low threshold, small volume solar pumped lasers. This optical pumping scheme is general, and applicable to solar down-shifting and optical gain materials in the visible and infrared portions of the solar spectrum. Cascade energy transfer^10^ enables large Stokes shifts between solar absorption and optical gain emission, a necessary condition for very-low-threshold daylight-pumped lasers as outlined by Roxio and Yablonovitch^15^.

As a proof of principle, we constructed a solar-pumped laser based on a conventional 1% atomic Nd^3+^ doped Yittrium Aluminum Garnet (YAG) gain medium that we ground and polished to form a planar waveguide with length, width and thickness of 43 mm × 2 mm × 750 μm, respectively. The Nd^3+^:YAG waveguide was coated with a 15-μm-thick poly(vinyl butyral-co-vinyl alcohol-co-vinyl acetate) (PVB-CVA-CVAc) thin film containing 10% wt. colloidal CdSe/CdZnS nanocrystals, provided by QD Vision, Inc. The colloidal nanocrystal quantum efficiency (QE), defined as the ratio between the number of emitted and absorbed photons, is 75% when doped into PVB-CVA-CVAc, and the film is photostable in air. The overlap between the solar spectrum and the absorption of the CdSe/CdZnS:PVB-CVA-CVAc thin film can be seen in Fig. 2a, showing the broad spectral absorption of the nanocrystals between 350 nm and 600 nm. The narrow emission and tunability of the nanocrystal photoluminescence allows for efficient pumping of the narrow absorption peaks of Nd^3+^:YAG. Indeed, as shown in Fig. 2b, the CdSe/CdZnS photoluminescence is tuned to neodymium’s main absorption peak in the visible at 585 nm. A disadvantage of the chosen nanocrystal emitter, however, is the small Stokes shift between absorption and emission, which leads to reabsorption and eventually loss of the waveguided photoluminescence^16^. Self-absorption losses can be reduced in future device iterations through energy transfer methods^10^,^17^, Stokes shift engineered nanocrystals^18^,^19^, and optical techniques^20^ to reduce the overlap between absorption and emission of the luminescent emitter.

To measure the efficiency of cascade energy transfer from input light to excited Nd^3+^ ions, the Nd^3+^:YAG waveguide is illuminated by monochromatic light at various wavelengths (see METHODS) with and without a 15-μm-thick 10% wt. CdSe/CdZnS:PVB-CVA-CVAc thin film. To increase the absorption of the incident light within the sample, a simple backing silver mirror is employed. Luminescence from the sample is collected with an integrating sphere and 850 nm long pass wavelength filter is used to discriminate between CdSe/CdZnS and Nd^3+^:YAG emission. The key parameter is the efficiency of cascade energy transfer, which we define as the fraction of pump photon rate ultimately transferred to Nd^3+^. Cascade energy transfer depends on the nanocrystal absorption, photoluminescent quantum yield, the fraction of nanocrystal photoluminescence trapped in the waveguide, and the competition between photoluminescence reabsorption losses in the nanocrystal film and waveguided pumping of the optical gain media. As can be seen by the blue line in Fig. 3a, the poor absorption of isolated Nd^3+^:YAG sample in the visible spectrum leads to very little energy transfer from the pump to the Nd^3+^. With the addition of the thin film of CdSe/CdZnS:PVB-CVA-CVAc on the Nd^3+^:YAG waveguide, cascade energy transfer generates a broad visible excitation spectrum with peak value of 14% that follows the absorption profile of the nanocrystal (gray dotted line). Averaged over the solar spectrum out to the nanocrystal absorption cutoff at λ = 600 nm, the waveguide pump efficiency due to the nanocrystal is 8.3%, resulting in total pump efficiency of 9.7%; see grey dashed line and red line in Fig. 3a. For the bare cavity the pump efficiency at similar spectral range is only 2.35%. This efficiency enhancement by a factor of 4.1 demonstrates the transfer of optical power to small volume optical gain media via a broadly absorbing luminescent colloidal nanocrystal intermediate. A Monte Carlo ray tracing simulation was developed to model the effect of cascade energy transfer on the distribution of excited Nd^3+^ within the waveguide. The inputs to the simulation are the sample geometry, the material’s spectral absorption coefficients and refractive indices, photoluminescence spectrum, and photoluminescence quantum yield. An input elliptical Gaussian beamshape is used to simulate concentrated input light, with full width half max of FWHMW = 1 mm and FWHML = 26 mm along the width and length of the sample respectively. In Fig. 3b, we integrate along the length and the thickness of the Nd^3+^:YAG waveguide, and determine the density of excited Nd^3+^ ions per 100 μm width across the sample. Normalization of this parameter with the pump photon flux results in the relative distribution of Nd^3+^ excited states. This pump distribution analysis is done at three different pump wavelengths. Incident light at a wavelength of λ = 735 nm is beyond the absorption edge of the nanocrystals and only excites neodymium ions. The resultant profile of Nd^3+^ excited states mirrors the non-uniformity of the pump intensity distribution. Absorption of incident light at λ = 405 nm, however, is dominated by the nanocrystals. Subsequent cascade energy transfer produces a near uniform Nd^3+^ population despite the non-uniformity in incident intensity. The improved uniformity is a result of the relatively long absorption path length of waveguided photoluminescence compared to the sample thickness. Modelling predicts that 15.3% of incident photons at λ = 405 nm are transferred to Nd^3+^ as compared to the measured 14%. The slight discrepancy between simulation and experiment may be due to scattering at surfaces which is not included in the model. Finally, for pump wavelengths such as λ = 531 nm, that excite neodymium and the nanocrystals, the resultant excited state profile is a weighted average of direct absorption and cascade energy transfer. The modelled total integrated Nd^3+^ excited states for pump wavelength of λ = 531 nm is 7.4% compared to the measured 6.3%. These simulations indicate that cascade energy transfer results in improved uniformity of the excited state population and thermal dissipation within the optical gain media.

To determine the laser threshold, the sample is placed in a hemispherical resonator as shown in Fig. 1 and excited by a λ = 531 nm laser with spot size as defined in the Monte Carlo ray tracing simulation. We observe that the doped PVB-CVA-CVAc polymer films used in this study are thermally stable for CW pumping below 10 W-cm^−2^ without active cooling, as seen in the inset of Fig. 4b. The instability we observe at 10 W-cm^−2^ is due to the low glass transition temperature Tg ~ 75 °C 21 of the host used. Consequently, we employ a quasi-CW scheme with pump pulse widths of approximately 10 ms to eliminate the possibility of thermal degradation of the down-shifting films. We observe both spectral narrowing of the main Nd^3+^ transition at λ = 1064 nm, as depicted in Fig. 4a, and threshold behavior at an incident power of 7.9 W. Taking into account the illumination distribution, as simulated in Fig. 3b, the maximal local flux of such pump at threshold is 14.54 W-cm^−2^. Figure 4b shows the threshold characterization in terms of maximal local flux. This induces maximal local population inversion of N_t_ = 8.45 × 10^21^ Nd^3+^-m^−3^. Because the excitation distribution occupies only part of the sample, a conservative estimation of the 531 nm pump that is required for lasing at the entire sample would be equal to maximal photon flux of 14.54 W-cm^−2^. Calculating the solar concentration required to induce the same maximal flux must take into account the difference between the pumping efficiency at 531 nm (6.3%) and the average pumping efficiency over the solar spectrum between 350 nm and 825 nm (6.8%; see red line in Fig. 3a). In addition, because only 46% of the total solar flux is within this wavelength range the overall solar pumping efficiency is 3.12%, leading to the required solar flux of 23 W-cm^2^ or 230 suns. Averaging the pump contribution of the NCs (gray line in Fig. 3a) over the solar spectrum shows that in the case of Nd^3+^ excitations generated only by NC emission, the laser will operate at pump threshold of 415 suns.

The lasing threshold, efficiency, and stability of the cascade energy transfer scheme can be improved through three methods: (i) enhanced solar absorption with near-infrared (NIR) rather than visible spectrum colloidal nanocrystals^22^, (ii) higher efficiency waveguide pumping using colloidal nanocrystals with lower self-absorption through an increased Stokes shift^18^,^19^ and higher photoluminescence quantum yield, and (iii) smaller mode volume optical gain media^23^. The dependence of the laser threshold on the properties of the luminescent concentrator can be seen in Fig. 5. By moving to a NIR absorbing and emitting colloidal nanocrystal, the amount of photons available to the gain material would double, reducing the gain threshold by a factor of two. However, a larger benefit is expected from improving the waveguide pump efficiency, plotted on the x-axis in Fig. 5, which can be increased by improving the in-film photoluminescence quantum yield of the colloidal nanocrystals and optimizing the Stokes shift to reduce self-absorption of waveguided photoluminescence. Such improvements should not only result in a reduced threshold power, but should also result in a reduction in heat generation within the down-shifting film. The improvement of waveguide pumping efficiency and solar absorption in the NIR could result in significant reduction in solar concentration threshold from this proof of principal system.

In conclusion, cascade energy transfer presents a path to efficient solar powered lasers operating under passive cooling, and potentially in the non-tracking regime^24^. The pairing of luminescent colloidal nanocrystals with traditional optical gain media such as Nd^3+^ and Tm^3+^ allows the decoupling of solar absorption from mode volume and enables solar laser operation from the visible to the near infrared. Solar powered Tm^3+^ lasers are especially notable because they potentially enable the efficient up-conversion of sub-bandgap infrared sunlight for silicon solar cells.

## Methods

The Nd^3+^:YAG slab waveguide was formed through conventional polishing techniques from commercial laser rods of diameter 3 mm and length of 43 mm with λ = 1064 nm antireflection coatings on both faces. Organic soluble CdSe/CdZnS colloidal semiconductor nanocrystals that emit at 585 nm were provided by QD Vision, Inc. The PVB-CVA-CVAc host was purchased from Sigma Aldrich. A solution of 100 mg/mL 10% wt. CdSe/CdZnS:PVB-CVA-CVAc in chloroform was drop cast onto the Nd^3+^:YAG waveguide producing a film of 15 μm thickness. The nanocrystal doped polymer film on glass was excited at λ = 405 nm and the photoluminescence spectrum was recorded using a calibrated Ocean Optics spectrometer positioned perpendicular to the substrate. The Nd^3+^:YAG and Nd^3+^:YAG with CdSe/CdZnS: PVB-CVA-CVAc film was excited with monochromatic light. An evaporated silver mirror was used behind the sample to double the optical path length of incident light. The monochromatic light was generated using a tungsten bulb, monochromator with a spectral resolution of 3.5 nm full width at half maximum, and a mechanical chopper. The sample photoluminescence was measured with a lock-in amplifier, spectrally calibrated photodetector, and long wavelength filters with a cutoff at λ = 850 nm were used to discriminate between Nd^3+^:YAG and CdSe/CdZnS emission in the integrating sphere. Although the fine structure of the Nd^3+^ absorption is partly obscured by the resolution of the monochromator, its spectral resolution does not affect calculations of the equivalent solar threshold obtained by integrating the product of solar and device excitation spectra. The laser resonator is a hemispherical cavity with a planar mirror of reflectivity 99.8% and an output mirror with a radius of curvature of 15 cm and reflectivity of 98%. A 18 W λ = 531 nm CW laser was used to optically excite the NC film coated Nd^3+:^YAG slab in air. An Acton spectrometer with a grating of 1000 grooves per mm and a calibrated Newport photodetector and neutral density filter were used to measure the lasing spectrum and power threshold of the laser respectively.

## Additional Information

**How to cite this article**: Reusswig, P. D. *et al.* A path to practical Solar Pumped Lasers via Radiative Energy Transfer. *Sci. Rep.* **5**, 14758; doi: 10.1038/srep14758 (2015).

## Figures and Tables

**Figure 1:**
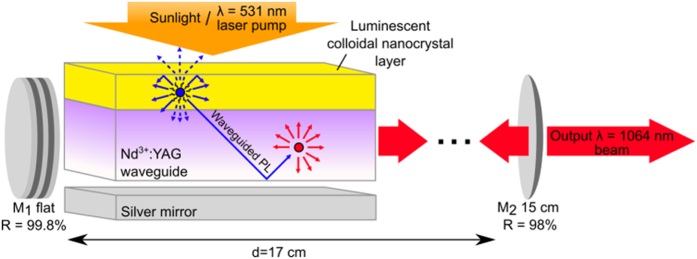
Illustration of cascade energy transfer for solar pumped lasers. A luminescent colloidal nanocrystal film (yellow) is optically coupled to the planar optical gain waveguide (purple) (not to scale). Incident sunlight is absorbed by the luminescent layer and largely re-emitted into waveguide modes. As the luminescence propagates in the waveguide, energy is transferred to the optical gain media through optical absorption. The optical gain media is then placed into a hemispherical resonator for a solar pumped laser via cascade energy transfer.

**Figure 2:**
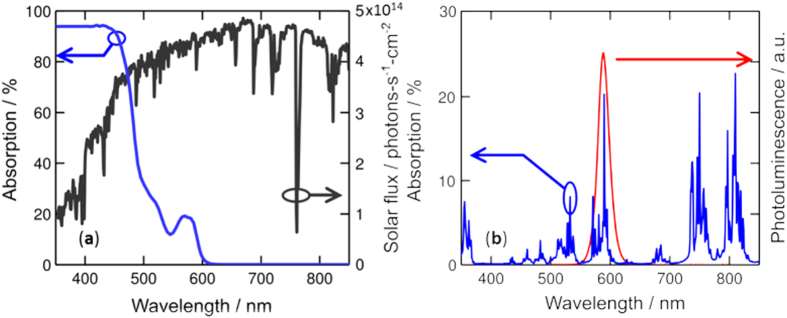
(**a**) Measured absorption of the luminescent colloidal film, 10% wt. CdSe/CdZnS:PVB-CVA-CVAc (blue), overlaid on the AM1.5 solar flux (black). Sunlight can be efficiently absorbed by the colloidal nanocrystals due to their broad and large absorption coefficients. (**b**) The luminescence of the nanocrystal (red) is tuned to an absorption peak of the Nd^3+^:YAG (blue), for efficient waveguided luminescent pumping of optical gain media.

**Figure 3:**
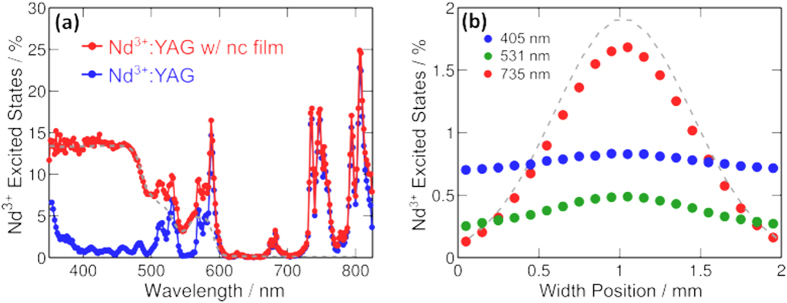
(**a**) Excitation spectra of a 750-μm-thick Nd^3+^:YAG crystal (blue), and the same crystal with a 10% wt. CdSe/CdZnS:PVB-CVA-CVAc coating (red). Scaled double pass absorption of the colloidal nanocrystal film is shown as a dashed gray line. (**b**) Modelled Nd^3+^ excited state distribution in the Nd^3+^:YAG coated waveguide for incident photons of wavelength λ = 405 (blue dots), 531 (green dots), and 735 (red dots)nm from the Monte Carlo ray tracing simulation. The pump photon spatial distribution is shown as a dashed gray line.

**Figure 4:**
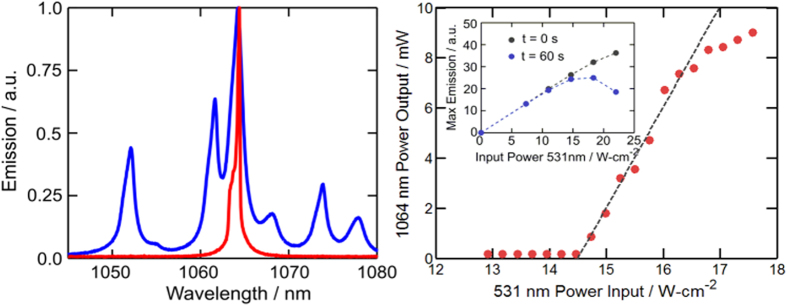
(**a**) Emission spectrum below (blue) and above (red) threshold. (**b**) Lasing power at λ = 1064 nm versus pump intensity showing a lasing threshold at 14.54 W-cm^−2^ local pump intensity. Inset showing nanocrystal photoluminescence versus pump intensity at t = 0 min and t = 1 min. Above 10 W-cm^−2^, the steady-state stability of the polymer doped film is reduced. A quasi-CW pump was used for lasing experiments.

**Figure 5:**
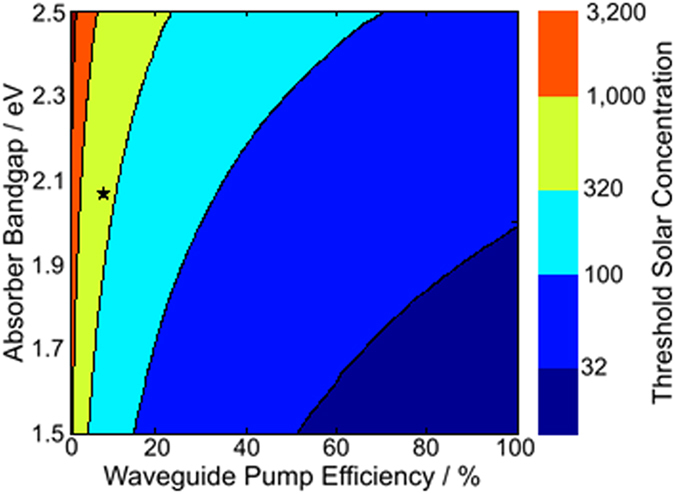
The calculated lasing threshold of the presented system as a function of the absorption edge of the luminescent colloidal nanocrystals and the efficiency of energy transfer to the gain medium, disregarding direct solar absorption by Nd^3+^. The measured equivalent threshold solar concentration due to cascade energy transfer reported in this work is marked as a star.
